# Supplementary material for: SPIRIT-CONSORT-TM: a corpus for assessing transparency of clinical trial protocol and results publications
Source: Sci Data. 2025 Feb 28;12:355. doi: 10.1038/s41597-025-04629-1 (PMC11871027; doi:10.1038/s41597-025-04629-1)
Supplement: Supplementary file 1 — Supplementary Tables [file 41597_2025_4629_MOESM1_ESM.docx]

### Supplementary Information for *SPIRIT-CONSORT-TM: a corpus for assessing transparency of clinical trial protocol and results publications*

Lan Jiang, Colby J Vorland^,^ Xiangji Ying, Andrew W Brown, Joe D Menke, Gibong Hong, Mengfei Lan, Evan Mayo-Wilson, and Halil Kilicoglu

We provide token-level statistics for the training, validation, and test splits of the SPIRIT-CONSORT-TM corpus, along with item-level details, and item-level performance of the NLP models below.

Table S1 presents the token-level statistics of the dataset across training, validation, and test splits.

| **Split** | **Total no. of tokens** | **Mean (SD)** | **Median (IQR)** |
| --- | --- | --- | --- |
| Training | 854,289 | 6,102.06 (1,953.11) | 5,918.00 (2,003.50) |
| Validation | 108,870 | 5,443.50 (1,094.30) | 5,695.00 (1,434.25) |
| Test | 247,948 | 6,198.70 (1,870.28) | 6,098.00 (1,621.50) |

**Table S1**. Token-level descriptive statistics of SPIRIT-CONSORT-TM. SD: standard deviation.

Table S2 presents descriptive statistics of the dataset at the sentence and article levels.

| **Checklist Item** | **No. of articles** | **Avg. number of sentences per article (SD)** | **Range** |
| --- | --- | --- | --- |
| 1a_Title_Randomized | 168 | 0.85 (0.39) | 0-2 |
| 1b_Title_Type | 33 | 0.17 (0.37) | 0-1 |
| 1c_Title_Framework | 17 | 0.09 (0.28) | 0-1 |
| 1d_Title_Centers | 19 | 0.10 (0.29) | 0-1 |
| 1e_Title_Population | 188 | 0.95 (0.26) | 0-2 |
| 1f_Title_Intervention | 189 | 0.95 (0.24) | 0-2 |
| 1g_Title_Acronym | 68 | 0.34 (0.47) | 0-1 |
| 2_Abstract_structured | 184 | 0.92 (0.27) | 0-1 |
| 3a_Registry_number | 195 | 1.44 (0.68) | 0-5 |
| 3b_Protocol_access** | 90 | 0.81 (1.15) | 0-6 |
| 4_Funding | 188 | 2.71 (2.54) | 0-20 |
| 5a_Sponsor | 30 | 0.21 (0.55) | 0-3 |
| 5b_Contributors_roles | 153 | 5.22 (4.59) | 0-36 |
| 5c_Oversight_committees | 43 | 1.52 (9.03) | 0-87 |
| 7_Objectives | 198 | 2.85 (2.48) | 0-13 |
| 8a_Design_Type | 105 | 0.70 (0.83) | 0-5 |
| 8b_Design_Framework | 134 | 1.12 (1.15) | 0-6 |
| 8c_Design_Centers | 177 | 1.35 (0.87) | 0-4 |
| 8d_Design_Ratio | 148 | 0.99 (0.79) | 0-4 |
| 9_Setting | 186 | 2.22 (3.33) | 0-31 |
| 10a_Participants_inclusion | 200 | 5.70 (6.18) | 1-40 |
| 10b_Center_interventionist_inclusion | 50 | 0.58 (1.56) | 0-11 |
| 11a_Intervention_Description | 199 | 20.36 (19.74) | 0-122 |
| 11b_Intervention_Modification | 45 | 0.50 (1.44) | 0-14 |
| 11c_Intervention_Monitoring | 124 | 2.62 (4.97) | 0-45 |
| 11d_Intervention_Concomitant | 104 | 1.63 (3.78) | 0-36 |
| 12a_Outcomes_Definitions | 198 | 19.15 (19.42) | 0-116 |
| 12b_Outcomes_Changes** | 11 | 0.09 (0.44) | 0-4 |
| 13_Participant_timeline | 103 | 1.85 (5.33) | 0-60 |
| 14a_Sample_size | 183 | 1.42 (1.13) | 0-11 |
| 14b_Sample_Calculation | 186 | 5.44 (6.35) | 0-44 |
| 15_Recruitment | 143 | 2.51 (3.51) | 0-26 |
| 16a_Randomization_Generation | 143 | 0.78 (0.54) | 0-2 |
| 16b_Randomization_Type | 142 | 0.99 (0.87) | 0-6 |
| 16c_Randomization_Block_size | 73 | 0.40 (0.56) | 0-2 |
| 16d_Randomization_Strata | 104 | 0.62 (0.68) | 0-3 |
| 16e_Allocation_Mechanism | 127 | 0.76 (0.89) | 0-10 |
| 16f_Allocation_Concealment | 133 | 1.10 (1.22) | 0-9 |
| 16g_Personnel_Sequence | 119 | 0.70 (0.72) | 0-5 |
| 16h_Personnel_Enrollment | 96 | 0.74 (1.00) | 0-6 |
| 17a_Masking_People_masked | 134 | 1.20 (1.23) | 0-7 |
| 17b_Masking_Not_masked | 93 | 0.56 (0.68) | 0-3 |
| 17c_Masking_Type | 109 | 0.78 (0.93) | 0-5 |
| 17d_Masking_Unblinding | 22 | 0.21 (0.96) | 0-9 |
| 17e_Masking_Similarity | 55 | 0.49 (0.99) | 0-5 |
| 18a_Data_Collection | 174 | 7.97 (11.44) | 0-84 |
| 18b_Data_Retention | 76 | 0.87 (1.55) | 0-9 |
| 19_Data_Management | 79 | 1.43 (3.13) | 0-23 |
| 20a_Statistical_methods_Outcomes | 196 | 7.71 (7.43) | 0-48 |
| 20b_Statistical_methods_Other_Analyses | 101 | 1.52 (2.79) | 0-19 |
| 20c_Statistical_methods_Analysis_population | 143 | 1.35 (1.35) | 0-8 |
| 20d_Statistical_methods_Missing_data | 89 | 0.74 (1.12) | 0-7 |
| 21a_Data_monitoring_committee | 58 | 0.88 (2.26) | 0-20 |
| 21b_Interim_analyses | 42 | 0.52 (1.79) | 0-19 |
| 21c_Stopping_guidelines | 28 | 0.40 (2.38) | 0-32 |
| 22_Harms_non-systematic | 85 | 1.55 (4.16) | 0-39 |
| 23_Auditing* | 18 | 0.27 (1.01) | 0-7 |
| 24_Ethics | 193 | 1.83 (2.48) | 0-32 |
| 25_Amendments | 47 | 0.53 (1.44) | 0-14 |
| 26a_Consent_Obtaining | 194 | 2.40 (2.89) | 0-27 |
| 26b_Consent_Provisions* | 4 | 0.04 (0.27) | 0-3 |
| 27_Confidentiality | 72 | 0.85 (1.83) | 0-14 |
| 28_Financial_interests | 170 | 2.62 (4.46) | 0-46 |
| 29_Data_access* | 24 | 0.19 (0.60) | 0-4 |
| 30_Post_trial_care* | 16 | 0.17 (0.77) | 0-8 |
| 31a_Dissemination | 37 | 0.57 (1.54) | 0-11 |
| 31b_Authorship | 14 | 0.12 (0.59) | 0-7 |
| 31c_Sharing_Materials | 24 | 0.23 (0.71) | 0-6 |
| 31d_Sharing_Data | 82 | 0.99 (1.55) | 0-13 |
| 31e_Sharing_Code | 7 | 0.05 (0.30) | 0-3 |
| 32_Informed_consent_materials | 8 | 0.05 (0.28) | 0-3 |
| 33_Biological_specimens | 14 | 0.29 (1.30) | 0-10 |
| 34_Flow** | 98 | 2.16 (2.74) | 0-12 |
| 35a_Recruitment_dates | 145 | 0.95 (0.78) | 0-4 |
| 35b_Followup_dates | 74 | 0.43 (0.61) | 0-3 |
| 35c_Stopping** | 15 | 0.11 (0.49) | 0-5 |
| 36_Baseline_data** | 100 | 2.46 (3.65) | 0-16 |
| 37a_Analysis_Numbers** | 82 | 0.79 (1.33) | 0-10 |
| 38a_Outcome_results** | 99 | 9.20 (13.11) | 0-70 |
| 38b_Binary_results** | 64 | 1.55 (4.11) | 0-44 |
| 39_Ancillary_results** | 76 | 3.31 (8.01) | 0-88 |
| 40_Harms_results** | 47 | 1.06 (2.87) | 0-23 |
| 41_Generalizability** | 81 | 1.07 (1.95) | 0-12 |

**Table S2**. Sentence-level descriptive statistics of checklist items in SPIRIT-CONSORT-TM. SD: standard deviation. *: SPIRIT-only items, **: CONSORT-only items.

Table S3 shows the descriptive statistics of the term-level dataset at the item level.

| **Checklist Item** | **No. of articles** | **No. of instances** | **Avg. frequency of terms per article (SD)** | **Range (# of annotations)** | **Avg. length of tokens (SD)** | **Range (token length)** |
| --- | --- | --- | --- | --- | --- | --- |
| 1a_Title_Randomized | 168 | 172 | 0.86 (0.40) | 0-2 | 1.02 (0.23) | 1-4 |
| 1b_Title_Type | 33 | 34 | 0.17 (0.39) | 0-2 | 1.47 (0.85) | 1-3 |
| 1c_Title_Framework | 17 | 20 | 0.10(0.35) | 0-2 | 1.15 (0.36) | 1-2 |
| 1d_Title_Centers | 19 | 19 | 0.10 (0.29) | 0-1 | 1.74 (0.91) | 1-3 |
| 1e_Title_Population | 188 | 210 | 1.05 (0.48) | 0-4 | 4.30 (3.06) | 1-18 |
| 1f_Title_Intervention | 189 | 245 | 1.23 (0.64) | 0-4 | 4.24 (2.99) | 1-16 |
| 1g_Title_Acronym | 68 | 68 | 0.34 (0.47) | 0-1 | 1.71 (1.09) | 1-7 |
| 3a_Registry_number | 195 | 304 | 1.52 (0.82) | 0-6 | 1.19 (1.00) | 1-13 |
| 8a_Design_Type | 105 | 153 | 0.77 (0.99) | 0-7 | 2.24 (1.77) | 1-11 |
| 8b_Design_Framework | 134 | 261 | 1.30 (1.54) | 0-10 | 2.94 (4.85) | 1-31 |
| 8c_Design_Centers | 177 | 279 | 1.40 (0.96) | 0-6 | 2.75 (2.01) | 1-17 |
| 8d_Design_Ratio | 148 | 206 | 1.03 (0.87) | 0-5 | 4.00 (2.10) | 2-14 |
| 14a_Sample_size | 183 | 357 | 1.78 (1.42) | 0-13 | 3.05 (3.24) | 1-29 |
| 16a_Randomization_Generation | 143 | 154 | 0.77 (0.54) | 0-2 | 4.55 (4.01) | 1-22 |
| 16b_Randomization_Type | 142 | 240 | 1.20 (1.06) | 0-6 | 1.49 (1.29) | 1-12 |
| 16c_Randomization_Block_size | 73 | 84 | 0.42 (0.60) | 0-3 | 3.48 (3.15) | 1-12 |
| 16d_Randomization_Strata | 104 | 218 | 1.09 (1.39) | 0-8 | 6.07 (7.16) | 1-43 |
| 17a_Masking_People_masked | 134 | 381 | 1.91 (2.04) | 0-13 | 2.15 (2.10) | 1-21 |
| 17b_Masking_Not_masked | 93 | 167 | 0.83 (1.09) | 0-6 | 2.08 (1.84) | 1-10 |
| 17c_Masking_Type | 109 | 157 | 0.79 (0.94) | 0-5 | 2.74 (0.88) | 1-9 |
| 20c_Statistical_methods_Analysis_population | 143 | 352 | 1.76 (2.01) | 0-16 | 7.28 (7.97) | 1-72 |
| 20d_Statistical_methods_Missing_data | 89 | 150 | 0.75 (1.14) | 0-7 | 7.89 (8.26) | 1-45 |

**Table S3.** Descriptive statistics regarding the annotation of term-level checklist items in SPIRIT-CONSORT-TM. SD: standard deviation.

Tables S4 and S5 show the NLP model performance over 5 runs at the sentence and article levels. 95% CIs are not included for brevity.

| **Checklist Item** | **Prec.** | **Recall** | **F1** |
| --- | --- | --- | --- |
| 1a_Title_Randomized | 0.899 | 1.000 | 0.946 |
| 1b_Title_Type | 1.000 | 0.500 | 0.667 |
| 1c_Title_Framework | 0.000 | 0.000 | 0.000 |
| 1d_Title_Centers | 1.000 | 0.933 | 0.960 |
| 1e_Title_Population | 0.897 | 0.979 | 0.936 |
| 1f_Title_Intervention | 0.917 | 0.959 | 0.937 |
| 1g_Title_Acronym | 0.986 | 0.943 | 0.964 |
| 3a_Registry_number | 0.900 | 0.949 | 0.923 |
| 3b_Protocol_access | 0.824 | 0.766 | 0.793 |
| 4_Funding | 0.894 | 0.906 | 0.900 |
| 5a_Sponsor | 0.767 | 0.680 | 0.708 |
| 5b_Contributors_roles | 0.984 | 0.963 | 0.973 |
| 5c_Oversight_committees | 0.584 | 0.700 | 0.635 |
| 7_Objectives | 0.835 | 0.860 | 0.845 |
| 8a_Design_Type | 0.929 | 0.439 | 0.593 |
| 8b_Design_Framework | 0.598 | 0.621 | 0.608 |
| 8c_Design_Centers | 0.633 | 0.512 | 0.566 |
| 8d_Design_Ratio | 0.822 | 0.657 | 0.730 |
| 9_Setting | 0.801 | 0.611 | 0.693 |
| 10a_Participants_inclusion | 0.908 | 0.874 | 0.891 |
| 10b_Center_interventionist_inclusion | 0.500 | 0.227 | 0.303 |
| 11a_Intervention_Description | 0.807 | 0.910 | 0.855 |
| 11b_Intervention_Modification | 0.240 | 0.100 | 0.133 |
| 11c_Intervention_Monitoring | 0.591 | 0.543 | 0.561 |
| 11d_Intervention_Concomitant | 0.687 | 0.337 | 0.451 |
| 12a_Outcomes_Definitions | 0.787 | 0.672 | 0.724 |
| 12b_Outcomes_Changes** | 0.000 | 0.000 | 0.000 |
| 13_Participant_timeline | 0.447 | 0.530 | 0.481 |
| 14a_Sample_size | 0.732 | 0.646 | 0.685 |
| 14b_Sample_Calculation | 0.876 | 0.855 | 0.865 |
| 15_Recruitment | 0.554 | 0.737 | 0.632 |
| 16a_Randomization_Generation | 0.881 | 0.893 | 0.887 |
| 16b_Randomization_Type | 0.818 | 0.844 | 0.828 |
| 16c_Randomization_Block_size | 0.969 | 0.871 | 0.917 |
| 16d_Randomization_Strata | 0.910 | 0.657 | 0.761 |
| 16e_Allocation_Mechanism | 0.581 | 0.539 | 0.556 |
| 16f_Allocation_Concealment | 0.587 | 0.594 | 0.588 |
| 16g_Personnel_Sequence | 0.642 | 0.627 | 0.632 |
| 16h_Personnel_Enrollment | 0.343 | 0.353 | 0.344 |
| 17a_Masking_People_masked | 0.857 | 0.816 | 0.836 |
| 17b_Masking_Not_masked | 0.797 | 0.520 | 0.626 |
| 17c_Masking_Type | 0.926 | 0.610 | 0.729 |
| 17d_Masking_Unblinding | 0.400 | 0.400 | 0.394 |
| 17e_Masking_Similarity | 0.661 | 0.346 | 0.453 |
| 18a_Data_Collection | 0.390 | 0.527 | 0.447 |
| 18b_Data_Retention | 0.845 | 0.473 | 0.606 |
| 19_Data_Management | 0.737 | 0.609 | 0.666 |
| 20a_Statistical_methods_Outcomes | 0.713 | 0.675 | 0.692 |
| 20b_Statistical_methods_Other_Analyses | 0.603 | 0.565 | 0.582 |
| 20c_Statistical_methods_Analysis_population | 0.783 | 0.694 | 0.735 |
| 20d_Statistical_methods_Missing_data | 0.785 | 0.639 | 0.703 |
| 21a_Data_monitoring_committee | 0.844 | 0.507 | 0.633 |
| 21b_Interim_analyses | 0.913 | 0.392 | 0.548 |
| 21c_Stopping_guidelines | 0.764 | 0.919 | 0.832 |
| 22_Harms_non-systematic | 0.863 | 0.836 | 0.848 |
| 23_Auditing* | 0.383 | 0.600 | 0.465 |
| 24_Ethics | 0.872 | 0.881 | 0.876 |
| 25_Amendments | 0.878 | 0.560 | 0.677 |
| 26a_Consent_Obtaining | 0.782 | 0.734 | 0.757 |
| 26b_Consent_Provisions* | N/A | N/A | N/A |
| 27_Confidentiality | 0.752 | 0.686 | 0.716 |
| 28_Financial_interests | 0.952 | 0.957 | 0.954 |
| 29_Data_access* | 0.755 | 0.767 | 0.744 |
| 30_Post_trial_care | 0.333 | 0.050 | 0.084 |
| 31a_Dissemination | 0.819 | 0.900 | 0.857 |
| 31b_Authorship | 0.828 | 0.533 | 0.640 |
| 31c_Sharing_Materials | 0.400 | 0.200 | 0.266 |
| 31d_Sharing_Data | 0.750 | 0.929 | 0.829 |
| 31e_Sharing_Code | N/A | N/A | N/A |
| 32_Informed_consent_materials | 0.000 | 0.000 | 0.000 |
| 33_Biological_specimens | 0.058 | 0.200 | 0.090 |
| 34_Flow** | 0.705 | 0.861 | 0.775 |
| 35a_Recruitment_dates | 0.818 | 0.842 | 0.830 |
| 35b_Followup_dates | 0.841 | 0.533 | 0.653 |
| 35c_Stopping | 0.800 | 0.266 | 0.400 |
| 36_Baseline_data | 0.816 | 0.683 | 0.741 |
| 37a_Analysis_Numbers | 0.306 | 0.261 | 0.271 |
| 38a_Outcome_results | 0.802 | 0.846 | 0.824 |
| 38b_Binary_results | 0.361 | 0.529 | 0.427 |
| 39_Ancillary_results | 0.760 | 0.481 | 0.588 |
| 40_Harms_results | 0.912 | 0.892 | 0.901 |
| 41_Generalizability | 0.469 | 0.433 | 0.450 |
| Micro-Average | 0.756 | 0.729 | 0.742 |
| Macro-Average | 0.702 | 0.624 | 0.645 |

**Table S4**. Item-level performance of the sentence classification model for each item. Items with NA for performance did not have any instances in the test set.

| **Checklist Item** | **Prec.** | **Recall** | **F1** |
| --- | --- | --- | --- |
| 1a_Title_Randomized | 0.952 | 1.000 | 0.975 |
| 1b_Title_Type | 1.000 | 0.500 | 0.667 |
| 1c_Title_Framework | 0.000 | 0.000 | 0.000 |
| 1d_Title_Centers | 1.000 | 0.933 | 0.960 |
| 1e_Title_Population | 0.949 | 0.979 | 0.964 |
| 1f_Title_Intervention | 0.954 | 0.979 | 0.966 |
| 1g_Title_Acronym | 0.986 | 0.943 | 0.964 |
| 2_Abstract_structured | 0.971 | 0.971 | 0.971 |
| 3a_Registry_number | 0.974 | 0.995 | 0.984 |
| 3b_Protocol_access | 0.931 | 0.941 | 0.936 |
| 4_Funding | 0.979 | 0.984 | 0.982 |
| 5a_Sponsor | 0.767 | 0.760 | 0.737 |
| 5b_Contributors_roles | 1.000 | 0.933 | 0.966 |
| 5c_Oversight_committees | 0.762 | 0.785 | 0.769 |
| 7_Objectives | 0.979 | 0.944 | 0.961 |
| 8a_Design_Type | 0.948 | 0.615 | 0.743 |
| 8b_Design_Framework | 0.887 | 0.740 | 0.806 |
| 8c_Design_Centers | 0.856 | 0.662 | 0.745 |
| 8d_Design_Ratio | 0.930 | 0.810 | 0.865 |
| 9_Setting | 0.924 | 0.794 | 0.854 |
| 10a_Participants_inclusion | 1.000 | 0.975 | 0.987 |
| 10b_Center_interventionist_inclusion | 0.580 | 0.433 | 0.489 |
| 11a_Intervention_Description | 0.989 | 0.909 | 0.947 |
| 11b_Intervention_Modification | 0.327 | 0.249 | 0.279 |
| 11c_Intervention_Monitoring | 0.732 | 0.774 | 0.745 |
| 11d_Intervention_Concomitant | 0.660 | 0.609 | 0.631 |
| 12a_Outcomes_Definitions | 1.000 | 0.985 | 0.992 |
| 12b_Outcomes_Changes** | 0.000 | 0.000 | 0.000 |
| 13_Participant_timeline | 0.654 | 0.664 | 0.657 |
| 14a_Sample_size | 0.905 | 0.837 | 0.869 |
| 14b_Sample_Calculation | 0.967 | 1.000 | 0.983 |
| 15_Recruitment | 0.734 | 0.843 | 0.783 |
| 16a_Randomization_Generation | 0.963 | 0.911 | 0.936 |
| 16b_Randomization_Type | 0.929 | 0.918 | 0.922 |
| 16c_Randomization_Block_size | 0.969 | 0.938 | 0.953 |
| 16d_Randomization_Strata | 1.000 | 0.733 | 0.844 |
| 16e_Allocation_Mechanism | 0.705 | 0.597 | 0.642 |
| 16f_Allocation_Concealment | 0.816 | 0.745 | 0.776 |
| 16g_Personnel_Sequence | 0.686 | 0.793 | 0.735 |
| 16h_Personnel_Enrollment | 0.480 | 0.640 | 0.546 |
| 17a_Masking_People_masked | 1.000 | 0.924 | 0.960 |
| 17b_Masking_Not_masked | 0.931 | 0.564 | 0.703 |
| 17c_Masking_Type | 1.000 | 0.822 | 0.900 |
| 17d_Masking_Unblinding | 0.800 | 0.433 | 0.560 |
| 17e_Masking_Similarity | 0.749 | 0.531 | 0.619 |
| 18a_Data_Collection | 0.812 | 0.843 | 0.826 |
| 18b_Data_Retention | 0.880 | 0.715 | 0.785 |
| 19_Data_Management | 0.892 | 0.982 | 0.933 |
| 20a_Statistical_methods_Outcomes | 0.974 | 0.949 | 0.961 |
| 20b_Statistical_methods_Other_Analyses | 0.602 | 0.537 | 0.564 |
| 20c_Statistical_methods_Analysis_population | 0.902 | 0.842 | 0.869 |
| 20d_Statistical_methods_Missing_data | 0.924 | 0.708 | 0.800 |
| 21a_Data_monitoring_committee | 0.901 | 0.880 | 0.888 |
| 21b_Interim_analyses | 0.800 | 0.571 | 0.667 |
| 21c_Stopping_guidelines | 0.734 | 0.800 | 0.759 |
| 22_Harms_non-systematic | 0.875 | 0.789 | 0.829 |
| 23_Auditing* | 0.383 | 0.500 | 0.427 |
| 24_Ethics | 0.973 | 1.000 | 0.986 |
| 25_Amendments | 0.889 | 0.750 | 0.809 |
| 26a_Consent_Obtaining | 0.983 | 0.937 | 0.960 |
| 26b_Consent_Provisions* | N/A | N/A | N/A |
| 27_Confidentiality | 0.903 | 0.775 | 0.833 |
| 28_Financial_interests | 1.000 | 0.977 | 0.989 |
| 29_Data_access* | 0.750 | 0.800 | 0.755 |
| 30_Post_trial_care | 0.300 | 0.090 | 0.133 |
| 31a_Dissemination | 0.863 | 0.857 | 0.856 |
| 31b_Authorship | 0.767 | 0.600 | 0.662 |
| 31c_Sharing_Materials | 0.400 | 0.200 | 0.266 |
| 31d_Sharing_Data | 0.953 | 1.000 | 0.975 |
| 31e_Sharing_Code | N/A | N/A | N/A |
| 32_Informed_consent_materials | 0.000 | 0.000 | 0.000 |
| 33_Biological_specimens | 0.350 | 0.400 | 0.371 |
| 34_Flow** | 0.796 | 1.000 | 0.885 |
| 35a_Recruitment_dates | 0.881 | 0.926 | 0.902 |
| 35b_Followup_dates | 0.850 | 0.660 | 0.741 |
| 35c_Stopping | 0.800 | 0.266 | 0.400 |
| 36_Baseline_data | 0.918 | 1.000 | 0.957 |
| 37a_Analysis_Numbers | 0.400 | 0.435 | 0.406 |
| 38a_Outcome_results | 0.990 | 1.000 | 0.995 |
| 38b_Binary_results | 0.711 | 0.983 | 0.820 |
| 39_Ancillary_results | 0.695 | 0.759 | 0.725 |
| 40_Harms_results | 0.896 | 0.933 | 0.913 |
| 41_Generalizability | 0.636 | 0.710 | 0.671 |
| Micro-Average | 0.887 | 0.845 | 0.865 |
| Macro-Average | 0.799 | 0.744 | 0.761 |

**Table S5**. Article-level performance of the sentence classification model for each item. Items with NA for performance did not have any instances in the test set.

Table S6 shows the results of the term recognition model at the item level. 95% CIs are not included for brevity.

| **Checklist Item** | **Strict** | | | **Lenient** | | |
| --- | --- | --- | --- | --- | --- | --- |
|  | Prec. | Recall | F1 | Prec. | Recall | F1 |
| 1a_Title_Randomized | 1.000 | 1.000 | 1.000 | 1.000 | 1.000 | 1.000 |
| 1b_Title_Type | 1.000 | 0.500 | 0.667 | 1.000 | 0.500 | 0.667 |
| 1c_Title_Framework | 1.000 | 0.250 | 0.400 | 1.000 | 0.250 | 0.400 |
| 1d_Title_Centers | 1.000 | 0.333 | 0.500 | 1.000 | 0.333 | 0.500 |
| 1e_Title_Population | 0.514 | 0.475 | 0.494 | 0.892 | 0.825 | 0.857 |
| 1f_Title_Intervention | 0.568 | 0.532 | 0.550 | 0.857 | 0.766 | 0.809 |
| 1g_Title_Acronym | 0.846 | 0.786 | 0.815 | 0.923 | 0.857 | 0.889 |
| 3a_Registry_number | 0.873 | 0.965 | 0.917 | 0.889 | 0.983 | 0.933 |
| 8a_Design_Type | 0.611 | 0.393 | 0.478 | 0.706 | 0.429 | 0.533 |
| 8b_Design_Framework | 0.264 | 0.434 | 0.329 | 0.337 | 0.528 | 0.412 |
| 8c_Design_Centers | 0.568 | 0.438 | 0.494 | 0.595 | 0.458 | 0.518 |
| 8d_Design_Ratio | 0.774 | 0.585 | 0.667 | 0.903 | 0.683 | 0.778 |
| 14a_Sample_size | 0.521 | 0.576 | 0.547 | 0.548 | 0.606 | 0.576 |
| 16a_Randomization_Generation | 0.409 | 0.300 | 0.346 | 0.895 | 0.567 | 0.694 |
| 16b_Randomization_Type | 0.629 | 0.512 | 0.564 | 0.800 | 0.651 | 0.718 |
| 16c_Randomization_Block_size | 0.714 | 0.357 | 0.476 | 0.857 | 0.429 | 0.571 |
| 16d_Randomization_Strata | 0.500 | 0.303 | 0.377 | 0.737 | 0.424 | 0.538 |
| 17a_Masking_People_masked | 0.544 | 0.595 | 0.568 | 0.701 | 0.726 | 0.713 |
| 17b_Masking_Not_masked | 1.000 | 0.050 | 0.095 | 1.000 | 0.050 | 0.095 |
| 17c_Masking_Type | 0.838 | 0.775 | 0.805 | 0.865 | 0.800 | 0.831 |
| 20c_Statistical_methods_Analysis_population | 0.410 | 0.333 | 0.368 | 0.513 | 0.417 | 0.460 |
| 20d_Statistical_methods_Missing_data | 0.500 | 0.273 | 0.353 | 0.722 | 0.394 | 0.510 |
| OVERALL | 0.585 | 0.532 | 0.557 | 0.712 | 0.633 | 0.670 |

**Table S6**. Item-level performance from our term extraction model (PURE model with section header and relative position extension). Note that this table provides the item-level result from a single run; therefore the overall results differ from those in Table 4 of the main manuscript.
